# BDNF-Val66Met variant and adolescent stress interact to promote susceptibility to anorexic behavior in mice

**DOI:** 10.1038/tp.2016.35

**Published:** 2016-04-05

**Authors:** M Madra, L M Zeltser

**Affiliations:** 1 Institute of Human Nutrition, Columbia University, New York, NY, USA; 2 Naomi Berrie Diabetes Center, Columbia University, New York, NY, USA; 3 Department of Pathology and Cell Biology, Columbia University, New York, NY, USA

## Abstract

There is an urgent need to identify therapeutic targets for anorexia nervosa (AN) because current medications do not impact eating behaviors that drive AN's high mortality rate. A major obstacle to developing new treatments is the lack of animal models that recapitulate the pattern of disease onset typically observed in human populations. Here we describe a translational mouse model to study interactions between genetic, psychological and biological risk factors that promote anorexic behavior. We combined several factors that are consistently associated with increased risk of AN—adolescent females, genetic predisposition to anxiety imposed by the *BDNF*-Val66Met gene variant, social isolation stress and caloric restriction (CR). Approximately 40% of the mice with all of these risk factors will exhibit severe self-imposed dietary restriction, sometimes to the point of death. We systematically varied the risk factors outlined above to explore how they interact to influence anorexic behavior. We found that the Val66Met genotype markedly increases the likelihood and severity of abnormal feeding behavior triggered by CR, but only when CR is imposed in the peri-pubertal period. Incidence of anorexic behavior in our model is dependent on juvenile exposure to social stress and can be extinguished by adolescent handling, but is discordant from anxiety-like behavior. Thus, this study characterized gene × environment interactions during adolescence that could be the underlying driver of abnormal eating behavior in certain AN patients, and represents a promising system to identify possible targets for therapeutic intervention.

## Introduction

Anorexia nervosa (AN) is a complex multi-factorial disease characterized by a compulsive restriction of food intake, resulting in severe weight loss.^1, 2^ It is the third-most common chronic illness among US adolescent females,^3^ with lifetime prevalence estimates of 0.3–0.9% in females and 0.1–0.3% in males.^4, 5^ There is an urgent need to develop treatments for AN, because it has the highest mortality rate of any psychiatric disease.^6^ Due to AN's high co-morbidity with anxiety and depression,^7, 8, 9^ the efficacy of psychotropic medications such as serotonin-specific reuptake inhibitors (SSRIs) in AN patients has been examined by several different groups. While data are discordant regarding the benefits of SSRIs in reducing depressive symptoms, these treatments consistently have no impact on the restrictive eating behavior that is responsible for the high mortality.^10, 11, 12^

The medical community has struggled to understand what drives the relentless suppression of food intake in AN patients. Several factors have hindered efforts to gain mechanistic insights into proximal causes of self-imposed malnutrition associated with AN. While many genetic, biological, psychological and sociocultural variables appear to contribute significantly to susceptibility to AN, few are specific to AN, and no single factor has been shown to be either necessary or sufficient to induce the disorder.^8, 13, 14^ Functional imaging of ill or recovered AN patients has revealed alterations in activity or neurotransmitter binding in corticolimbic structures.^13, 15, 16, 17^ However, as most studies are performed months or years after disease onset, it is hard to distinguish primary causes of AN from secondary consequences of malnutrition on brain structure and function. Moreover, as many AN patients have a co-morbid psychiatric disorder and/or are taking SSRIs,^12^ it is not possible to parse those changes that contribute to disordered eating specifically. While model systems are usually helpful in these circumstances, the critical involvement of psychosocial risk factors has been an impediment to the development of reliable animal models of AN that approximate the human disease.^18, 19^

Genetic studies point toward familial aggregation of AN,^20^ and twin studies of AN support a high degree (~50%) of heritability.^21^ As none of the candidate gene variants identified thus far is sufficient to cause anorexia,^22, 23^ it is likely that these variants confer vulnerability to other environmental factors that ultimately lead to the pathological eating behavior. Interestingly, several genes that correlate with increased risk of AN in some patient cohorts have also been linked to a wide range of anxiety disorders.^24, 25^ As the likelihood and severity of AN have been linked to pre-existing anxiety disorders,^9, 14, 26, 27^ it has been suggested that a genetic predisposition to anxiety is a key mediator of susceptibility to AN.^20^

Brain-derived neurotrophic factor (BDNF) plays an important role in the development of neuronal circuits regulating cognitive-, anxiety- and eating-related behaviors.^28, 29, 30^ The common *BDNF*-Val66Met gene variant, characterized by impaired BDNF release and function,^29, 30, 31^ is associated with increased likelihood and severity of AN in some cohorts,^27, 32, 33^ as well as increased anxiety-like behaviors in mice and humans.^25, 30, 34^ There is a growing appreciation that the *BDNF*-Val66Met variant plays a critical role in mediating the influence of early-life stress on the severity of anxiety and depressive symptoms in adolescence.^35, 36, 37^ Although the *BDNF-*Val66Met variant and pre-existing anxiety are independently associated with increased severity of AN,^9, 26, 27^ the possibility that interactions between genetic and environmental factors increase the risk of abnormal eating behaviors has not been examined.

Dieting behavior is strongly associated with AN,^14, 38^ but as these studies are retrospective, it is hard to discern whether it is a trigger or symptom of the psychiatric condition.^14^ Although dieting is usually deliberate, AN may also be precipitated by unintentional weight loss, such as that associated with mourning, some medications or surgery.^39^ Several lines of evidence support the idea that acute caloric restriction (CR) interacts with psychological and genetic factors to promote anorexic behavior. There is extensive crosstalk between circuits regulating stress and feeding.^40^ Chronic activation of the central stress response system can suppress food intake in animal models^41, 42, 43^ and affective disorders in humans are often accompanied by changes in eating habits or body weight.^1^ In addition, the Val66Met allele has been implicated in the effects of severe CR to promote unhealthy eating behaviors.^44^

In this manuscript, we outline our efforts to develop a translational mouse model to study gene × environment interactions that promote susceptibility to anorexic behavior. This model incorporates genetic, psychological and biological variables that are implicated by epidemiological studies. We used mice that segregate for at least one copy of the human *BDNF*-Val66Met allele, which has been associated with increased likelihood and severity of AN in some cohorts,^27^ as well as increased anxiety-like behaviors in mice and humans.^25, 30^ To incorporate the pre-existing anxiety and/or depression that is common in AN,^9, 14, 26, 27^ we exposed mice to social isolation stress during adolescence, a condition that elicits behavioral and neuroendocrine phenotypes similar to those observed in mouse models of anxiety or depression.^45^ Moreover, there is precedence for interactions between this environmental stressor and genetic risk factors for other psychiatric disorders.^46^

Females with a genetic susceptibility to anxiety (conveyed by the *hBDNF-*Val66Met allele) were exposed to social isolation stress during adolescence. To simulate the impacts of dieting, we restricted caloric intake by 20–30% for 11 days. Approximately 40% of the female *hBDNF*-Val66Met carriers exposed to early social isolation stress and CR during adolescence exhibit severe self-imposed dietary restriction, sometimes to the point of death. By systematically varying the combination and timing of the risk factors outlined above, we are beginning to understand how they interact to influence the likelihood and severity of anorexic behavior. Our studies reveal that the timing and nature of early-life experiences dictate the penetrance and severity of anxiety and feeding endophenotypes observed in Val66Met carriers.

## Materials and methods

### Animals

All mice were generated from intercrosses of *hBDNF*^Val/Met^ mice,^31^ kindly provided by Joseph Gogos (Columbia University, New York, NY, USA). Animals were housed in temperature controlled rooms at 21 °C and subject to a 12 h light–dark cycle. Mice had ad libitum access to standard chow diet (Lab Diet: PicoLab Rodent Diet 5053) and water, unless otherwise indicated. Animals were randomly assigned to be either group housed (3–5 mice per cage) or singly housed from 5 weeks of age. Food hoppers were given to all singly housed animals to monitor caloric intake from 6 weeks of age. Animals were randomly assigned to be either ad libitum fed or exposed to a 20–30% reduction in caloric intake for 10–11 consecutive days starting at 7 weeks of age. For the restricted group, 70–80% of the caloric intake of controls was provided in two daily allotments. Food intake and body weight were assessed three times per week starting at 7 weeks of age. Animals were excluded if they were singly housed prior to 5 weeks of age or displayed abnormal feeding behaviors prior to 7 weeks of age. Adrenal glands were dissected after death and weighed to calculate adrenal/body weight ratio. Investigators were not blinded to groups. All procedures were performed within the guidelines of the Institutional Animal Care and Use Committee at the Columbia University Health Sciences Division.

### Genotyping

Initially, genotyping of the *hBDNF* locus was performed using PCR on DNA extracted from tail tips as described.^31^ To overcome technical difficulties sometimes encountered with this protocol, we developed a new primer set BDNF-F: 5′-TCCACCAGGTGAGAAGAGTGA-3′, and BDNF-R: 5′-GAGGCTCCAAAGGCACTTGA-3′, followed by restriction-enzyme analysis with *Bsa*A1, which cleaves the Val allele.

### Locomotor activity

Central vs peripheral locomotor activity was assessed through a photobeam-based activity monitoring system incorporated in the Indirect Calorimetry System combined with Feeding Monitor and TSE ActiMotsystem. Animals were allowed to acclimate for at least 24 h to the room in which the apparatus was located. Anxiety-related behavior was assessed for the first 24 h in the system in all groups.

### Corticosterone

Baseline serum for corticosterone was collected from tail bleeds on minimally stressed animals at 1000 hours in unheparinized tubes and allowed to clot before centrifugation, decanting and storage at −20 °C until use. Serum was analyzed for corticosterone content via RIA (MP Biomedicals, Orangeburg, NY, USA) in the laboratory of S. Wardlaw (Columbia University).

### Gene expression

Expression analyses were performed on the rostral third of the hypothalamus and pituitary using real-time quantitative PCR. Tissue samples were quickly dissected, snap frozen in liquid nitrogen and stored at −80 °C until the mRNA were extracted using RNeasy Plus Universal Mini Kit (Qiagen, Austin, TX, USA) according to the manufacturer's guidelines. Complementary DNA (cDNA) was synthesized by reverse transcription of total RNA using the Transcriptor First Strand cDNA Synthesis Kit (Roche, Mannheim, Germany). Expression of corticotrophin-releasing hormone (*Crh*) (forward, 5′-ATCTCACCTTCCACCTTCTGCG-3′; reverse, 5′-CCCGATAATCTCCATCAGTTTCC-3′) and Proopiomelanocortin (*Pomc*) (forward, 5′-AGTGCCAGGACCTCACCA-3′; reverse 5′-CAGCGAGAGGTCGAGTTTG-3′) were quantified on a LightCycler (Roche) using the LightCycler 480 SYBR Green I Master system (Roche). *Beta actin* (forward, 5′-AAGGAAGGCTGGAAAAGAGC-3′; reverse, 5′-AAATCGTGCGTGACATCAA-3′) was used as a housekeeping gene. Relative quantification was calculated using the 2−ΔΔCt formula where Ct is the cycle threshold at which the amplified PCR product was detected and 2−ΔΔCt represents the fold change in gene expression normalized to beta actin and relative to the control group.

### Adolescent handling

Animals exposed to peri-pubertal handling enrichment (singly housed *hBDNF*^Met/?^ females (GEH) and singly housed *hBDNF*^Met/?^ females exposed to CR (GEDH)) were held daily for ~3 min from week 6 (P42) to week 7 (P49). Animals were returned to their home cages after handling. Animals that did not receive handling were not disturbed from week 6 to 7.

### Statistics

The sample sizes in our study were chosen based on common practice in animal behavior experiments (10–15 animals per group). Sample sizes in singly housed *hBDNF*^Met/?^ females not exposed to CR (GE) and exposed to CR (GED)groups were initially powered to permit separate analyses of homozygotes (*hBDNF*^Met/Met^) and heterozygotes (*hBDNF*^Val/Met^). As previously reported,^30, 47^ we found that all phenotypes assessed were the same in *hBDNF*^Met/Met^ and *hBDNF*^Met/Val^ females (data not shown). Thus, we combined data from these genotypes into one group (*hBDNF*^Met/?^) for statistical analyses, creating groups with larger samples sizes than the others (GE *n*=34 and GED *n*=36). We compared the average number of aphagic episode (AE) per animal in each group, as opposed to the percent of animals with AEs in each group, because it provided a parametric value for statistical analyses. Statistical comparisons were performed between groups using two-tailed, unpaired Student's *t*-test or one-way analysis of variance (ANOVA) with Fisher's Protected Least Significant Difference (PLSD) *post hoc* analysis. A *P*-value less than 0.05 was considered to be statistically significant. Data are presented as group mean±s.e.m.

## Results

### Gene × environment interactions promote aphagic behavior

We first asked whether feeding-related behaviors in a mouse knock-in model of the *hBDNF*-Val66Met allele^31^ are impacted by persistent exposure to social isolation stress from 5 weeks of age, an environmental condition reported to modulate stress axis responsivity, behavior and body weight.^45, 46^ We focused our initial efforts on characterizing phenotypes in females, due to the increased prevalence of affective, anxiety and eating disorders.^48^ We studied four groups of mice: *hBDNF*^Val/Val^ (without the susceptibility allele) maintained in group housing (control (C)); *hBDNF*^Met/?^ (that is, homozygous or heterozygous for the Met allele) maintained in group housing (genetic susceptibility (G)); *hBDNF*^Val/Val^ singly housed from 5 weeks of age (environmental stressor (E)); and *hBDNF*^Met/?^ singly housed from 5 weeks (genetic susceptibility and environmental stressor (GE)) (Supplementary Table 1).

**Figure 1:**
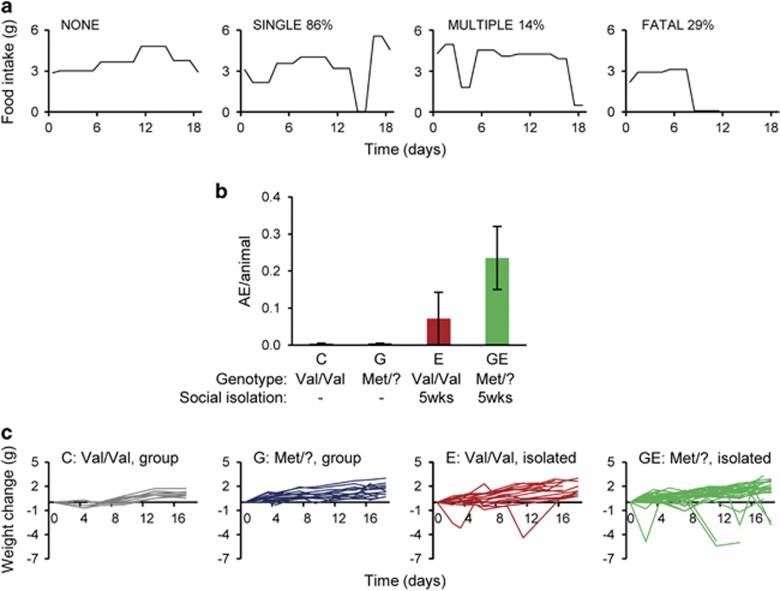
*hBDNF*^Met/?^ genotype interacts with adolescent social stress to promote abnormal feeding behavior. (**a**) Different patterns of daily food intake in *hBDNF*^Met/?^ females exposed to social isolation stress (GE). About 79.4% of animals maintained normal food intake throughout ‘NONE'. Of those that exhibited an AE, 85.7% exhibited only one aphagic episode (AE) ‘SINGLE', while 14.3% exhibited repeated AEs ‘MULTIPLE'. About 28.6% of the AEs resulted in death ‘FATAL'. Time 0 day starts at 7 weeks of age. (**b**) Number of aphagic episodes (AE) per animal per group from 7 to 9.5 weeks of age in group housed *hBDNF*^Val/Val^ (C) and *hBDNF*^Met/?^ (G) females; singly housed *hBDNF*^Val/Val^ (E) and *hBDNF*^Met/?^ (GE) females (C, 0 AE per animal, *n*=10, 2 cohorts; G, 0 AE per animal, *n*=13, 3 cohorts; E, 0.07±0.07 AE per animal, *n*=14, 3 cohorts; GE, 0.23±0.08 AE per animal *n*=34, 9 cohorts), ANOVA. (**c**) Body weight changes within respective groups, each line represents one animal. Time 0d starts at 7 weeks of age. (CTL, *n*=10; G, *n*=13; E, *n*=14; GE, *n*=34). Error bars denote s.e.m. ANOVA, analysis of variance; C, control; E, environmental stressor; G, genetic susceptibility; GE, genetic susceptibility and environmental stressor.

We measured food intake and body weight in 7-week-old females that were allowed to acclimate to feeding from hoppers for 1 week. We observed that a subset of the experimental animals completely refrained from eating for extended periods of time. Due to a variability of 0.5 g in measurements using food hoppers, we established a threshold of consumption of <0.5 g food over 24 h to quantify the incidence of ‘AEs'. AEs were typically followed by hyperphagia and a rebound to the initial body weight (Figure 1a, ‘SINGLE'). However, failure to resume eating in 28.6% (*n*=2/7) of aphagic mice was fatal (Figure 1a, ‘FATAL'). In addition, we found that 14.3% of singly housed *hBDNF*^Met/?^ females (GE) with AEs exhibited at least one additional bout of aphagia in the period analyzed (Figure 1a, ‘MULTIPLE').

Of *hBDNF*^Val/Val^ females exposed to social isolation stress (E) 7.1% exhibited AEs, while we did not observe any AEs in group housed *hBDNF*^Val/Val^ control (C) or *hBDNF*^Met/?^ (G) females (Figure 1b and Supplementary Table 1). Val66Met carriers exposed to social isolation stress (GE) exhibited a threefold increase in the prevalence of AEs compared with singly housed *hBDNF*^Val/Val^ (E), although this difference did not reach significance (Figure 1b and Supplementary Table 1). Of those mice that did not eat for 24 h, 87.5% remained aphagic for a second 24-h period and lost significant (>15%) body weight (Figure 1c).

### Dietary restriction can trigger abnormal feeding behavior

As we found that interactions between genetic factors and adolescent social stress increase the incidence of abnormal eating behavior, we next considered whether direct manipulations of caloric intake could also impact feeding behaviors in Val66Met carriers. Dieting behavior in adolescents usually precedes and has been proposed to act as a trigger of eating disorders.^38^ Moreover, the Val66Met allele has been implicated in the effects of severe CR to promote unhealthy eating behaviors.^44^ To explore this issue, we exposed singly housed *hBDNF*^Val/Val^ (E) or *hBDNF*^Met/?^ (GE) females to a mild dietary restriction (D) at 7 weeks, by providing them with 70–80% of the caloric intake of ad libitum-fed controls for 11 days, referred to as ED and GED groups, respectively (Supplementary Table 2). Due to technical limitations, we were unable to assess the impact of CR in the absence of social isolation stress (hypothetical groups ‘D' and ‘GD'). Because our mice could take over 3 h to consume all of their allotted food, there is no way to prevent one mouse from consuming another's food under group housed conditions.

**Figure 2:**
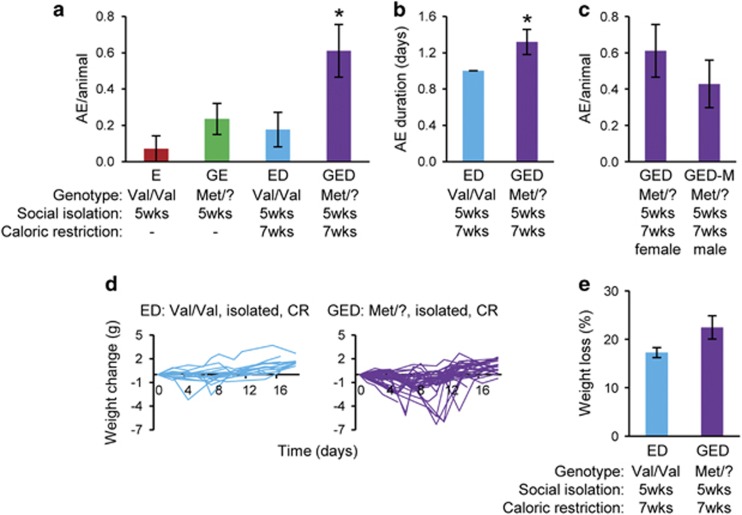
Peri-pubertal caloric restriction synergizes with genetic and environmental factors to promote abnormal feeding behavior. (**a**) Number of aphagic episodes (AE) per animal per group from 7 to 9.5 weeks of age in singly housed *hBDNF*^Val/Val^ (E) and *hBDNF*^Met/?^ (GE) females; or calorically restricted singly housed *hBDNF*^Val/Val^ (ED) and *hBDNF*^Met/?^ (GED) females (E, 0.07±0.07 AE per animal, *n*=14, 4 cohorts; GE, 0.23±0.08 AE per animal, *n*=34, 9 cohorts; ED, 0.18±0.95 AE per animal, *n*=17, 6 cohorts; GED, 0.61±0.14 AE per animal, *n*=36, 6 cohorts). *P<0.05, ANOVA. (**b**) Duration of AEs in ED and GED groups (ED, 1day, *n*=2; GED, 1.3±0.1 days, *n*=15). *P<0.05, Student's *t*-test. (**c**) Number of AE per animal per group triggered by caloric restriction in singly housed *hBDNF*^Met/?^ females (GED) and males (GED-M) (GED, 0.61±0.14 AE per animal, *n*=36, 6 cohorts; GED-M, 0.43±0.13 AE per animal, *n*=21, 3 cohorts). *P*<0.05, Student's *t*-test. (**d**) Body weight changes within respective groups, each line represents one animal. Time 0 day starts at 7 weeks. (ED, *n*=17; GED, *n*=36). (**e**) Body weight loss in ED and GED groups during AEs (ED, 17.2±1.5 g, *n*=2; GED 22.5±6.3 g, *n*=7) *P*<0.05, Student's *t*-test. Error bars denote s.e.m. ANOVA, analysis of variance; D, dietary stressor; E, environmental stressor; GE, genetic susceptibility and environmental stressor.

In the absence of genetic susceptibility factors, we observed an increased incidence of AEs in singly housed *hBDNF*^Val/Val^ females subjected to CR as compared with those fed ad libitum (0.18 AE per animal in ED vs 0.07 AE per animal in E) (Figure 2a and Supplementary Table 2), but this difference did not reach significance. Exposure to genetic, social and dietary risk factors was associated with a marked increase in the incidence of AEs compared with those groups exposed to any two risk factors (0.61 AE per animal in GED vs 0.18 AE per animal in ED vs 0.23 AE per animal in GE, *P*<0.05) (Figure 2a and Supplementary Table 2). Segregation for the Val66Met allele was also associated with an increase in the severity of AEs, as reflected in the average duration of an AE (1.3 days in GED vs 1.0 day in ED, *P*<0.05), and severity of weight loss (21.9% in GED vs 17.2% in ED) (Figures 2b, d and e). While the prevalence of AN is markedly higher in females,^5^ we found that the frequency of AEs in male Val66Met carriers that were exposed to adolescent social stress and CR (GED-M) was only 13% lower than in females, a difference that did not reach significance (Figure 2c). Together, these data support the idea that dieting can interact with genetic and environmental risk factors to promote abnormal feeding behavior.

### Vulnerability to peri-pubertal CR

**Figure 3:**
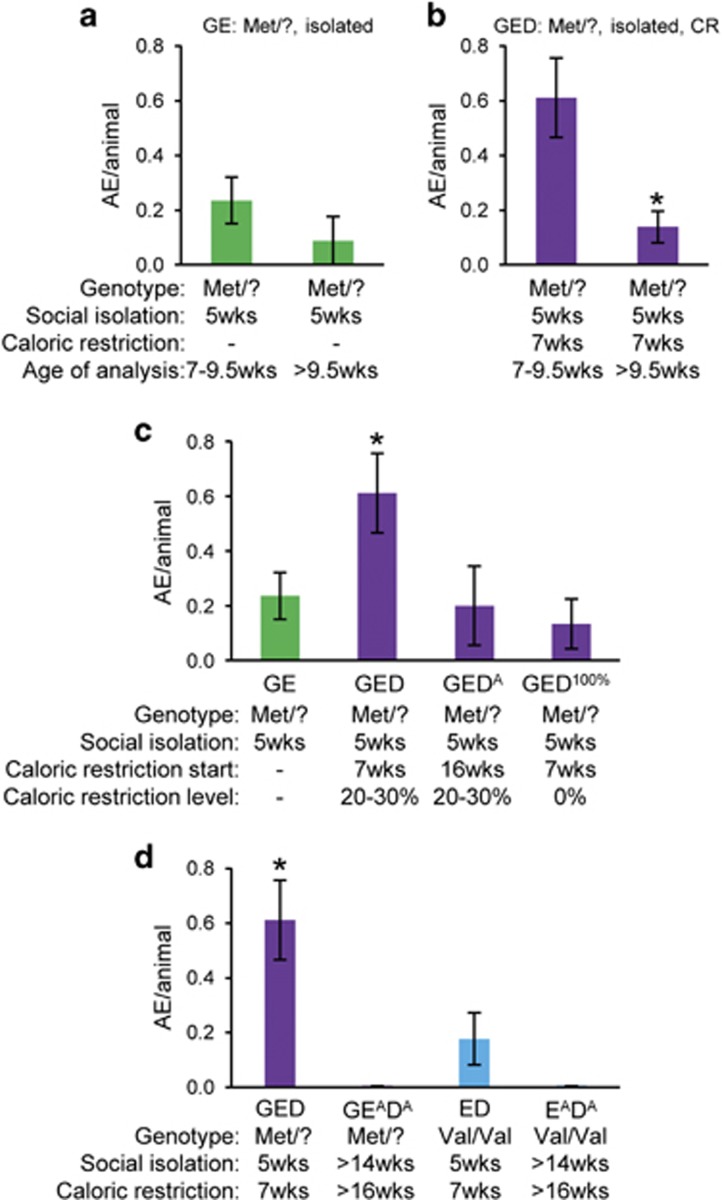
Impacts of caloric restriction and social isolation are conveyed in the peri-pubertal period. (**a**) Number of aphagic episodes (AE) per animal per group in singly housed *hBDNF*^Met/?^ females (GE) from 7–9.5 weeks of age compared with those observed after 9.5 weeks (GE: 7–9.5, 0.23±0.08 AE per animal; >9.5, 0.09±0.09 AE per animal, *n*=34, 9 cohorts). *P*<0.05, Student's *t*-test. (**b**) Number of AEs per animal per group in singly housed *hBDNF*^Met/?^ females under caloric restriction (CR) (GED) from 7–9.5 weeks of age compared with those observed after 9.5 weeks (GED: 7-9.5, 0.61±0.14 AE per animal; >9.5, 0.14±0.06 AE per animal, *n*=36, 6 cohorts). *P<0.05, Student's *t*-test. (**c**) Number of AEs per animal per group in GE, GED, singly housed *hBDNF*^Met/?^ females exposed to CR during adulthood (GED^A^) and singly housed *hBDNF*^Met/?^ females fed 100% of their daily caloric intake twice daily at 7 weeks (GED^100%^) (GE, 0.23±0.08 AE per animal, *n*=34, 9 cohorts; GED, 0.61±0.14 AE per animal, *n*=36, 6 cohorts; GED^A^, 0.20±0.14 AE per animal, *n*=15, 5 cohorts; GED^100%^, 0.13±0.09 AE per animal, *n*=15, 2 cohorts). *P<0.05, ANOVA. (**d**) Number of AEs per animal per group in GED, ED, *hBDNF*^Met/?^ and *hBDNF*^Val/Val^ females that were first exposed to single housing and CR during adulthood (GE^A^D^A^, E^A^D^A^ (GED, 0.61±0.14 AE per animal, *n*=36, 6 cohorts; GE^A^D^A^, 0 AE per animal, *n*=10, 2 cohorts; ED, 0.18±0.95 AE per animal, *n*=17, 6 cohorts; E^A^D^A^, 0 AE per animal, *n*=10, 2 cohorts). *P<0.05, ANOVA. Error bars denote s.e.m. ANOVA, analysis of variance; GE, genetic susceptibility and environmental stressor.

Eating disorders often emerge during adolescence after a period of intentional or unintentional weight loss.^4, 39^ Thus, we examined whether the timing of CR is critical. We compared food intake in females that were 7–9.5 weeks vs >9.5 weeks. The prevalence of AEs was markedly increased in the younger singly housed *hBDNF*^Met/?^ females with or without CR (GED and GE), although this difference only reached significance in the singly housed Val66Met carriers under CR (GED) group (0.61 AE per animal in GED <9.5 weeks vs 0.14 AE per animal in GED >9.5 weeks, *P*<0.05; Figures 3a and b). The lower incidence of AEs in GE and GED females older than 9.5 weeks was comparable to rates seen in young singly housed *hBDNF*^Val/Val^ females (0.07 AE per animal in E <9.5 weeks; Figure 1b).

Next, we compared the effect of exposing *hBDNF*^Met/?^ females that were singly housed from 5 weeks to an 11-day CR initiated in the peri-pubertal period (7 weeks=GED) vs adulthood (16 weeks of age= GED^A^). We observed that exposure to CR at 7 weeks was more than three times as likely to elicit an AE as compared with CR at 16 weeks (0.61 AE per animal in GED vs 0.2 AE per animal in GED^A^, *P*<0.05) (Figure 3c). Together, these findings support the idea that interactions between the *hBDNF*^Met/?^ genotype and CR that increase the likelihood of abnormal feeding behaviors are most pronounced in the peri-pubertal period, similar to observations regarding anxiety-like behavior.^49^

Twice-daily feeding regimens are associated with changes in neuronal circuits regulating energy balance,^50^ raising the possibility that psychological, rather than physiological responses to CR are acting in our model to promote abnormal feeding. To address this issue, we compared body weight and food intake phenotypes in singly housed Val66Met carrier females that were subjected to a twice-daily feeding protocol at 7 weeks with 100% (that is, no restriction) or 75% of the intake of ad libitum-fed controls (GED^100%^ and GED, respectively). We found that the incidence of AEs in GED^100%^ females that had limited access to 100% of the daily caloric intake was threefold lower than was observed in GED females exposed to 25% CR (0.13 AE per animal in GED^100%^ vs 0.61 AE per animal in GED, *P*<0.05; Figure 3c), and similar to that of singly housed Val66Met carriers that were not exposed to CR at all (0.24 AE per animal in GE; Figure 3c). These observations support the idea that physiological cues associated with reduced caloric intake contribute to the risk of abnormal feeding behaviors in our model.

### Impacts of social stress on susceptibility to AN-like behavior are conveyed during adolescence

Social stressors experienced during adolescence have been reported to synergize with genetic factors to influence discrete neurochemical and behavioral deficits observed in some affective disorders.^46^ To explore whether early exposure to social isolation stress is critical to elicit abnormal feeding behaviors, we compared the response to CR in adult females in which single housing was initiated at >14 weeks vs 5 weeks. We did not observe a single AE in singly housed *hBDNF*^Val/Val^ (E^A^D^A^) or *hBDNF*^Met/?^(GE^A^D^A^) females in response to CR when exposure to social isolation stress was started in adulthood (*n*=8–10 per group, Figure 3d).

### Anorexic behavior is not due to an exacerbation of anxiety-like behavior

**Figure 4:**
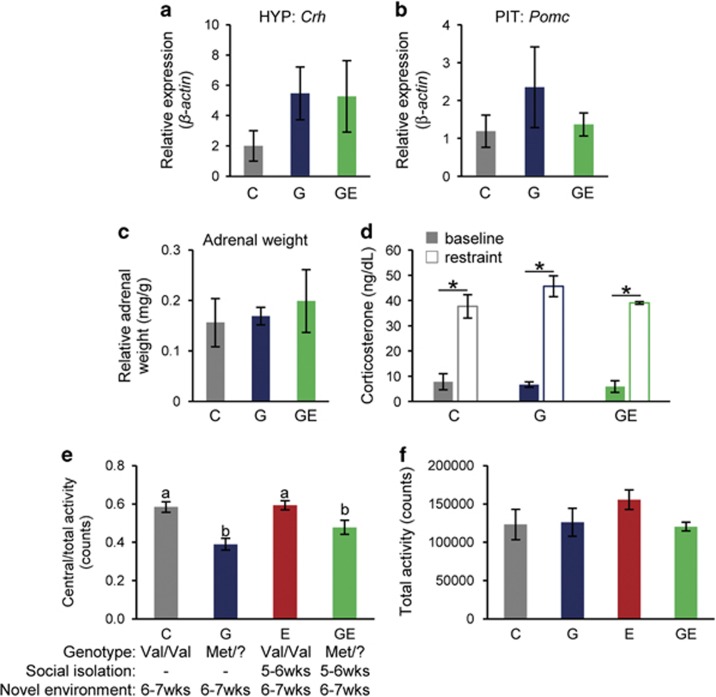
Anxiety-like behavior is increased in BDNF-Val66Met carriers, but is not exacerbated by exposure to social stress. (**a**) Expression, as measured by quantitative PCR, of *Corticosterone releasing hormone* (*Crh*) in the rostral 1/3 of the hypothalamus (HYP) (which contains the PVH) at 7 weeks in group housed, *hBDNF*^Val/Val^ (C) and *hBDNF*^Met/?^ (G) females, and singly housed *hBDNF*^Met/?^ (GE) females (C, 2.0±1.0 a.u., *n*=4, 1 cohort; G, 5.5±1.7 a.u., *n*=7, 1 cohort; GE, 5.3±2.4 a.u., *n*=8, 1 cohort). *P*<0.05, ANOVA. (**b**) Expression of *Proopiomelanocortin* (*Pomc*), as measured by quantitative PCR, in the pituitary (PIT) at 7 weeks in C, G and GE females (C, 1.2±0.4 a.u., *n*=4, 1 cohort; G, 0.17±0.02 a.u., *n*=3, 1 cohort; GE, 0.20±0.06 a.u., *n*=4, 2 cohorts). *P*<0.05, ANOVA. (**c**) Baseline and restraint corticosterone levels at 7 weeks in C, G and GE females (baseline: C, 7.8±3.2 a.u., *n*=6, 1 cohort; G, 6.7±1.0 a.u., *n*=7, 2 cohorts; GE, 5.9±2.3 a.u., *n*=6, 2 cohorts; restraint: C, 37.7±4.6 a.u., *n*=6, 1 cohort; G, 45.6±4.2 a.u., *n*=7, 2 cohorts; GE, 39.0±0.5 a.u., *n*=6, 2 cohorts). *P<0.05, ANOVA. (**d**) Weight of the adrenal glands, normalized to body weight, at 9.5 weeks in C, G and GE females (C, 0.16±0.05 mg g^−1^, *n*=5, 1 cohort; G, 2.3±1.1 mg g^−1^, *n*=7, 1 cohort; GE, 1.4±0.3 mg g^−1^, *n*=8, 1 cohort). *P*<0.05, ANOVA. (**e**) Normalized central activity counts in C, G, E and GE females at 6–7 weeks after exposure to a novel environment (C, 0.58±0.28 counts, *n*=3; G, 0.39±0.31 counts, *n*=7; E, 0.59±0.02 counts, *n*=3; GE, 0.48±0.04 counts, *n*=14, 3 distinct cohorts). ANOVA, lowercase letters above bars denote similar (*P*>0.05) groups. (**f**) Total activity at 6–7 weeks in C, G and GE females (C, 123198.3±19943.4 counts, *n*=3; G, 126131.7±18237.1 counts *n*=7; E, 155612.7±12822.76 counts, *n*=3; GE, 114137.8±8068.6 counts, *n*=14, 3 distinct cohorts). *P*<0.05, ANOVA. Error bars denote s.e.m. ANOVA, analysis of variance; C, control; E, environmental stressor; G, genetic susceptibility; GE, genetic susceptibility and environmental stressor; PVH, paraventricular nucleus of the hypothalamus.

The *BDNF*-Val66Met polymorphism has been associated with increased hypothalamus–pituitary–adrenal (HPA) axis reactivity and anxiety-like behaviors in mice and humans.^30, 34, 51, 52^ Therefore, we explored whether exacerbation of these phenotypes by social isolation could underlie the increased anorexic behavior in our model. We used a combination of molecular, neuroendocrine and physiological criteria to evaluate HPA axis function: expression of genes encoding *Crh* in the rostral hypothalamus (which contains the paraventricular nucleus of the hypothalamus) and *Pomc* in the pituitary, serum levels of the stress hormone CORT at baseline and in response to restraint stress and adrenal gland weights. We found that *Crh* and *Pomc* expression in group housed Val66Met carriers (G) at 7 weeks of age were more than twofold higher than in controls, although this difference did not reach significance (Figures 4a and b). Social isolation (GE) did not amplify these phenotypes (Figures 4a and b). We failed to detect significant effects of the Val66Met genotype (G) or social isolation (GE) on acute and chronic measures of HPA axis activity in any of the groups (Figures 4c and d). Mice in all groups (C, G, GE) exhibited similar adrenal weights (Figure 4d) and corticosterone (CORT) levels at baseline and in response to restraint stress (Figure 4c). Consistent with previous reports,^30^ we observed that Val66Met carriers exhibited increased anxiety-like behavior (0.39 central/total activity counts in G vs 0.58 central/total activity counts in C, *P*<0.05); however, this was not further exacerbated by social isolation (0.48 central per total activity counts in GE; Figures 4e and f). Together, these observations support the idea that gene × environment interactions that promote abnormal feeding behavior in our model are not correlated with further exacerbations of anxiety-like behavior already imposed by the *hBDNF-*Val66Met genotype.

### Adolescent handling prevents anorexic behavior

**Figure 5:**
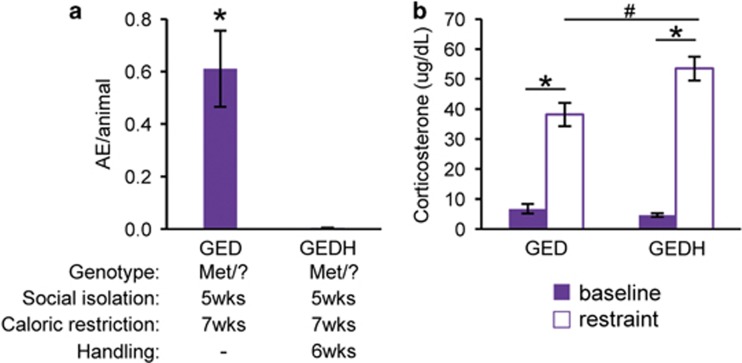
Daily adolescent handling prevents aphagic behavior, but increases HPA axis responsiveness. (**a**) Number of AEs per animal per group in singly housed *hBDNF*^Met/?^ females not exposed to daily handling (GED) and GED females exposed to daily handling for 1 week prior to CR (GEDH) (GED, 0.61±0.14 AE per animal, *n*=36, 6 cohorts; GEDH, 0 AE per animal, *n*=10, 2 cohorts). *P<0.05, Student's *t*-test. (**b**) Corticosterone levels at baseline (0 min) and after 15-min immobilization stress (restraint) in 9.5-week-old singly housed *hBDNF*^Met/?^ females exposed to caloric restriction (CR) (GED) and handling (GEDH) (baseline: GED, 6.75±1.59 μg dl^−1^, *n*=13, 3 cohorts; GEDH, 4.65±0.58 μg dl^−1^, *n*=12, 2 cohorts; restraint: GED, 38.20±3.88 μg dl^−1^, *n*=13, 3 cohorts; GEDH, 53.51±4.03 μg dl^−1^, *n*=12, 2 cohorts). *P<0.05, Student's *t*-test; baseline to restraint. ^#^*P*<0.05, Student's *t*-test; GED to GEDH. Error bars denote s.e.m. AE, aphagic episode; CR, caloric restriction; HPA, hypothalamus–pituitary–adrenal axis.

Handling has been shown to reverse the effects of social isolation stress on some behavioral and neuronal endpoints.^53, 54, 55^ In singly housed Val66Met carrier females that were handled ~3 min every day for the week preceding CR (GEDH), we did not observe a single AE (Figure 5a). We explored whether the beneficial effects of daily handling might be mediated via reductions in the neuroendocrine response to psychological stress. We assessed the response to immobilization for 15 min at 9.5 weeks, after release from CR. Daily handling of GED females from 6 to 7 weeks did not affect CORT levels at baseline (6.7 μg dl^−1^ in GED vs 4.6 μg dl^−1^ in GEDH *P*=0.23), but was unexpectedly associated with an elevated stress response at the end of the restraint period (38.2 μg dl^−1^ in GED vs 53.5 μg dl^−1^ in GEDH *P*<0.05) (Figure 5b). These findings argue against the possibility that the effect of handling to prevent anorexic behavior in GEDH mice is mediated through diminutions in HPA responsiveness.

## Discussion

We developed a model to test the hypothesis that the Val66Met allele increases the likelihood of AN by conferring sensitivity to environmental factors. We found that the Val66Met genotype promotes anorexic behavior in mice exposed to social isolation stress and CR during adolescence, but not when these environmental variables are imposed in adulthood. Gene × environment impacts on HPA axis function and anxiety-like behaviors were discordant with the incidence of anorexic behavior.

### A novel mouse model to study triggers of AN

Approaches involving genetic, environmental and/or dietary manipulations have been used by other labs to study AN in animal models. The *anx/anx* mouse strain carries a genetic mutation that leads to dramatic reductions in food intake and body weight from the postnatal period,^56^ which is a notable contrast to the common adolescent age of onset in humans.^4, 21^ Genetically engineered mouse strains have been used to ablate or activate distinct neuronal populations, with dramatic effects to suppress food intake.^57, 58, 59^ While these models have yielded novel insights into circuits that cause anorexia *per se*, it is not clear whether any given circuit contributes to the pathophysiology of AN in humans. Exposure to chronic and/or severe psychological stress directly suppresses food intake,^60^ however, the proximity of the timing and severity of the stressor are very different from the conditions that increase the risk of AN in humans.^8, 61^ The best-characterized animal model of AN is the activity-based anorexia model, which involves self-imposed starvation in response to exposure to a combination of restricted access to food and exercise.^62^ While these models have provided novel insights into neuropeptide and neuronal pathways responsible for food intake suppression, they have not yielded insights into the triggers of AN in humans.

While there will always be questions about the extent to which a mouse model can fully capture a disorder as complex as AN, several key aspects of self-imposed AEs in our model accurately reflect the conditions thought to promote eating disorders: (1) interactions between early-life stress and the *BDNF*-Val66Met genotype increase susceptibility; (2) onset is often preceded by dieting; and (3) peak incidence in adolescence.

While our model recapitulates many of the risk factors associated with susceptibility to AN,^4, 8, 20, 21, 61, 63^ there are two notable differences—lack of chronicity of self-imposed CR and diminished gender preference. During an AE in mice, food intake is suppressed by more than 80%. Thus, mice that maintain AEs for 3 days do not survive. Because the degree of food intake restriction is less severe in humans, this behavior can be maintained over a long period of time. It has been proposed that dieting and weight loss become a rewarding habit in some individuals, which fosters the persistence of this self-destructive behavior.^64^ Our observations are consistent with the idea that circuits responsible for triggering restrictive feeding behavior may be distinct from those that maintain it over a long period of time. If true, addressing both aspects of the pathological behavior may be needed to develop efficacious treatments for AN.

There are two factors that could contribute to the apparent discrepancy between the modest increase in AE prevalence in females in our model (Figure 2c) and epidemiological observations that males comprise only 10% of the AN patients in clinical and case registry studies.^4^ First, population-based studies estimate that the lifetime risk of AN is only threefold lower in males,^5^ supporting the idea that there are more subclinical cases of AN in males that go unreported. In addition, it is possible that the preponderance of AN in females is driven by gender differences in behavior—namely the propensity to diet—rather than physiology. Depending on the metric considered, the prevalence of unhealthy dieting behaviors is two to fourfold higher in females.^65^ As we observed that CR increases the likelihood of AN by twofold, the lower prevalence of dieting in adolescent boys could account for their reduced risk of AN.

### Implications for efforts to treat AN

Of the 11 experimental groups studied, 2 conditions were associated with resistance to anorexic behavior group housing during adolescence (control and G groups, Figure 1b) or daily handling in singly housed adolescents (GEDH, Figure 5a). Notably, both group housed Val66Met carriers (G group) and handled singly housed Val66Met carriers (GEDH group) exhibited increased behavioral (Figure 4e) and neuroendocrine (Figure 5b) responses to psychological stress. These observations are consistent with reports that enrichment paradigms improve behavioral responses in mice exposed to early-life stress.^66^ While the consequences of daily handling during the postnatal period have been described, less is known about the impacts during adolescence. Pharmacological treatments can reverse the impacts of adolescent social stress on the neuroanatomical organization of circuits in the prefrontal cortex.^67^ To explore whether compounds that modulate neurotransmitter activity might be used as therapies for AN, it is critical to identify the circuits responsible for anorexic behavior in our model. Our findings raise the possibility that behavioral or pharmacological therapeutic strategies aimed at mitigating antecedent exposure to juvenile stress, rather than co-morbid psychiatric symptoms, represent a promising approach to preventing and treating eating disorders.

### Future studies

In conclusion, our findings support the idea that exposure to juvenile social stress in genetically predisposed individuals increases the likelihood that CR in the peri-pubertal period will elicit anorexic behavior independent of effects on anxiety-related endpoints. The key lesson learned from our studies—that the timing and nature of early-life experiences dictate the penetrance and severity of psychological and feeding behaviors observed in Val66Met carriers—should inform efforts to close the ‘heritability gap' between estimates of the genetic contribution to AN based on classical twin studies, and the small and inconsistent effects often seen in association studies involving common gene variants. Going forward, understanding how developmental exposures interact with genetic variants to confer risks that cut across traditional diagnostic categories of mental illness could pave the way for the design of treatment strategies that are targeted to the gene × environment exposure, rather than a particular outcome measure or diagnostic category.
