# Supplementary material for: BDNF-Val66Met variant and adolescent stress interact to promote susceptibility to anorexic behavior in mice
Source: Transl Psychiatry. 2016 Apr 5;6(4):e776–. doi: 10.1038/tp.2016.35 (PMC4872394; doi:10.1038/tp.2016.35)
Supplement: Supplementary Tables [file tp201635x1.doc]

**Supporting Information:**

Supplementary Table 1

| **Group** | **n** | **Genetic (G)** | **Environmental (E)** | **Animals with AE (%)** | **Statistical analysis to GE** |
| --- | --- | --- | --- | --- | --- |
| **CTL** | 10 | BDNF^-Val/Val^ | Group housing | 0 | Fisher’s exact, 2-tail, p=0.18 |
| **G** | 13 | BDNF^-Met/?^ | Group housing | 0 | Fisher’s exact, 2-tail, p=0.17 |
| **E** | 14 | BDNF^-Val/Val^ | Single housing | 7.1 | Fisher’s exact, 2-tail, p=0.41 |
| **GE** | 34 | BDNF^-Met/?^ | Single housing | 20.6 | N/A |

Table S1. Experimental groups. Shaded boxes indicate manipulated factors different from control (CTL) in peri-pubertal female mice.

Supplementary Table 2

| **Group** | **n** | **Genetic (G)** | **Environmental (E)** | **Dieting (D)** | **Animals with AE (%)** | **Statistical Analysis to GED** |
| --- | --- | --- | --- | --- | --- | --- |
| **CTL** | 10 | BDNF^-Val/Val^ | Group Housing | Ad libitum Fed | 0 | Fisher’s exact, 2-tail, p=0.019 |
| **ED** | 17 | BDNF^-Val/Val^ | Single Housing | Caloric Restriction | 11.8 | Fisher’s exact, 2-tail, p=0.019 |
| **GED** | 36 | BDNF^-Met/?^ | Single Housing | Caloric Restriction | 41.7 | N/A |

Table S2. Experimental groups**.** Shaded boxes indicate manipulated factors different from control (CTL) in female peri-pubertal mice.
